# Clinicopathological Features and Prognosis of Primary GISTs with Tumor Rupture in the Real World

**DOI:** 10.1245/s10434-018-6505-7

**Published:** 2018-05-11

**Authors:** Toshirou Nishida, Haruhiko Cho, Seiichi Hirota, Toru Masuzawa, Gaku Chiguchi, Toshimasa Tsujinaka, the Kinki GIST Study Group, Kazuhiro Nishikawa, Tetsuji Sawada, Tsunehiro Maeda, Masaru Kawai, Hirofumi Ikushima, Kouji Nakai, Izumi Kawamoto, Syugo Ueda, Takuya Nakai, Masazumi Zaima, Shinsuke Sato, Kazuto Yajima, Tsuyoshi Takahashi, Hisashi Kubo, Kazumasa Fujitani, Junichi Hasegawa, Chikara Ebisu, Junya Fujita, Hiroshi Imamura, Atsushi Yamamoto, Noriyuki Yamamura, Tsunehiro Yoshimura, Mitsutoshi Tatsumi, Akinori Noguchi, Miki Noguchi, Hirokazu Matsuo, Akihiro Toyokawa, Seiichiro Kanaya, Takashi Yasuda, Takeshi Omori, Toru Masuzawa, Toshirou Nishida, Toshimasa Tsujinaka, Haruhiko Cho

**Affiliations:** 1 Department of Surgery, National Cancer Center Hospital, Tokyo, Japan; 2 Department of Surgery, Osaka Police Hospital, Osaka, Japan; 3 Present Address: Department of Surgery, Kansai Rosai Hospital, Amagasaki, Japan; 4 Department of Gastrointestinal Surgery, Kanagawa Cancer Center, Yokohama, Japan; 5 Present Address: Department of Surgery, Tokyo Metropolitan Cancer and Infectious Disease Center Komagome Hospital, Tokyo, Japan; 6 Department of Surgical Pathology, Hyogo College of Medicine, Nishinomiya, Japan; 7 Department of Gastroenterology, Yokohama Rosai Hospital, Yokohama, Japan; 8 Department of Gastrointestinal Surgery, Kaizuka City Hospital, Kaizuka, Japan

## Abstract

**Background:**

Patients with ruptured gastrointestinal stromal tumor (GIST) are recommended for imatinib adjuvant therapy; however, their clinicopathological features and prognosis in the era of imatinib are unknown.

**Patients and Methods:**

The study cohort included 665 patients with histologically proven primary GISTs who underwent R0 or R1 surgery between 2003 and 2007; the validation cohort included 182 patients between 2000 and 2014. The definitions of tumor rupture in the study included perforation at tumor site, tumor fracture, piecemeal resection including open biopsy, and macroscopic injuries to the pseudocapsule.

**Results:**

Tumor rupture occurred in 21 (3.2%) of 665 and 5 (2.9%) of 182 patients in the study and validation cohort, respectively. Ruptured GISTs were more symptomatic, were larger in size, and had higher mitotic count than nonruptured GISTs but were not associated with tumor location or laparoscopic surgery. GISTs with intraoperative rupture had clinicopathological features and prognostic outcomes similar to those with preoperative rupture. Recurrence rates were higher and median recurrence-free survival (RFS) and overall survival (OS) were shorter with ruptured than nonruptured GIST. Tumor rupture was one of the independent prognostic factors for RFS, but not OS, according to multivariate analysis.

**Conclusions:**

Ruptured GISTs were symptomatic larger tumors with high mitotic activity, frequent relapse, and shorter RFS. Tumor rupture was an independent prognostic factor for RFS, but not for OS, in the era of imatinib.

**Electronic supplementary material:**

The online version of this article (10.1245/s10434-018-6505-7) contains supplementary material, which is available to authorized users.

Gastrointestinal stromal tumor (GIST) is a potentially malignant tumor and the most frequent sarcoma of the gastrointestinal tract. Currently, complete resection (R0) of primary GIST offers the only potentially permanent cure for GIST patients. After complete resection, 60% of patients with localized GISTs are cured, while the other 40% experience disease recurrence during follow-up.^1^–^3^ Prognostic factors for recurrence after R0 surgery have been rigorously investigated, and four factors are recognized as independent prognostic factors: tumor size (cm), mitosis (/50 HPF or/5 mm^2^), location (gastric versus nongastric), and rupture.^3^–^13^ Several risk stratifications have been proposed based on these four factors.^4^–^8^

Among these four prognostic factors, tumor rupture is the most ominous and is a subjective factor clinically judged by surgeons.^3^,^10^–^12^ Previous reports indicate that most ruptured GISTs are associated with recurrence during follow-up and that patients with ruptured GIST have significantly shorter recurrence-free survival (RFS) than those without rupture.^14^–^16^ The definition of ruptured GIST, however, was not consistent among researchers until a recent provisional definition was proposed.^14^ The term “ruptured GIST” may include heterogeneous entities; hence, in some reports, rupture may include tumor penetration into the peritoneum (T4a) and microscopic pseudocapsule injuries. Incidence of rupture has been reported to vary from 2 to 22%.^3^,^7^,^10^,^12^,^16^–^18^ Some reports have suggested that tumor rupture is an independent prognostic factor,^3^,^5^,^10^,^12^,^16^ whereas others have disagreed.^19^,^20^

Recent clinical trials show that patients at high risk of recurrence may benefit from 3 years of adjuvant therapy with imatinib (Gleevec, Novartis Pharmaceuticals, Basel, Switzerland) after R0 resection.^17^,^18^ It may be argued that patients with ruptured GIST require prolonged adjuvant therapy for longer than 3 years. Few studies have examined which tumors are more likely to rupture or which surgical procedures, open or laparoscopic, may potentially increase the risk of rupture.^21^ In this retrospective study, we analyzed real-world data obtained from two registry studies conducted in Japan and clarified the clinicopathological features and prognostic effects of rupture in the era of imatinib.

## Patients and Methods

### Study Design

The study included both study and validation cohorts. The former data were obtained from the GIST registry study conducted by the Kinki GIST Study Group, and the latter from the Kanagawa registry study. The GIST registry designed by the Kinki GIST Study Group was reported previously.^22^ This retrospective observational study was designed to collect clinical and pathological data of patients with pathologically proven GIST diagnosed in each participating hospital and treated between January 2003 and December 2007. Data on risk stratifications classified according to the modified National Institutes of Health (NIH) consensus criteria^4^ and the Armed Forces Institute of Pathology (AFIP) criteria^6^ were collected, in addition to information regarding preoperative and intraoperative ruptures. The Kanagawa registry study collected similar patient data retrospectively and prospectively since January 2001 to December 2016 from the two hospitals in Kanagawa Prefecture. The hospitals participating in each registry are listed at the end of the manuscript.

Using data obtained from the GIST registry database, eligible patients were selected (Supplementary Fig. 1). Eligibility criteria included tumor morphology compatible with GIST, KIT positivity on immunohistochemistry, and macroscopically complete resection of the primary tumor. We excluded patients with metastatic or recurrent GIST at time of diagnosis and individuals whose date of surgery, age, gender, and/or outcomes were missing (Supplementary Fig. 1). This study was reviewed and approved by the Steering Committee of the Kinki GIST Study Group and by the institutional review board (IRB) of Osaka Police Hospital and Kanagawa Cancer Center.

Tumor rupture was considered to be subjectively determined by each surgeon at that time, so we conducted a retrospective survey of the concept of rupture by questionnaire. Questionnaire entries included perforation at the tumor site, tumor fracture with blood-tinged ascites, piecemeal resection during surgery (including open biopsy), and macroscopic injuries to the pseudocapsule exposing tumor cells into the peritoneal cavity (Supplementary Table 1). These were similar to the definitions of rupture proposed by Holmebakk et al.^14^ Completeness of surgical resection was assessed by local surgeons as follows: R0, no detectable residual tumor; R1, microscopic residual tumor; R2, macroscopic residual tumor. Patients with R2 resection were excluded from the analysis.

### Statistical Analysis

Statistical analyses were performed using the Chi squared test, Fisher’s exact test, Student’s *t* test, and Mann–Whitney *U* test. RFS was calculated from date of surgery to date of first tumor recurrence or date of death, excluding living patients without recurrence at time of data collection. Overall survival (OS) was calculated from date of surgery to date of any death, excluding living patients. RFS and OS were compared between the groups using the Kaplan–Meier life-table method with the log-rank test. Cumulative recurrence probability was estimated by cumulative incidence competing risk analysis as described previously.^23^ A forward stepwise Cox proportional hazards model was used for multivariate analysis to identify risk factors associated with RFS and OS. *P* values were two-sided, and *P* values less than 0.05 were considered significant. Data were analyzed using the Statistical Package of IBM SPSS Statistics 25, version 25.0 (IBM Corp., Armonk, NY, USA).

## Results

In total, 665 patients with primary GIST who underwent R0 or R1 surgery were included in the study cohort and 172 patients in the validation cohort. The clinicopathological features of the patients analyzed in the study and validation cohorts are presented in Table 1. The study cohort included 506 gastric, 119 small intestinal, 26 colorectal, 10 esophageal, and 4 extra-gastrointestinal GISTs. The study sample included 339 males and 326 females with median age of 66 years. Median tumor size was 4.0 cm, and the median mitotic count was 2.6/50 HPF; 21 (3.2%) patients were reported to have tumor rupture, either preoperatively (*N* = 12) or intraoperatively (*N* = 9). In the validation cohort, there were 120 gastric, 37 small intestinal, 14 colorectal, and 2 esophageal GISTs. Median age was 62.5 years, with 100 male and 72 female patients (Table 1). Median tumor size was 5.0 cm, the median mitotic count was 5.0/50 HPF, and only five patients (2.9%) had tumor rupture, of whom three had preoperative and two intraoperative rupture. Most patients in both cohorts underwent R0 surgery, by either open or laparoscopic procedure. Nearly all patients received imatinib therapy after relapse. A small fraction of patients with high-risk features received neoadjuvant (1.3 and 8.7%), while a relatively larger number of patients received adjuvant therapy (5.6 and 17.4%) in the study and validation cohort, respectively.

**Table 1:** Patient characteristics

Factor		Study cohort (*N* = 665)	Validation cohort (*N* = 172)
Age	(Median; years)	66.0 (18–93)	62.5 (17–89)
Gender	Male	339 (51.0%)	100 (58.1%)
	Female	326 (49.9%)	72 (41.9%)
Cancer association	Cancer history	113 (17.0%)	37 (21.5%)
	No cancer history	536 (80.6%)	135 (78.5%)
	Unavailable	16 (2.4%)	0 (0%)
Location	Esophagus	10 (1.5%)	2 (1.2%)
	Stomach^b^	506 (76.1%)^b^	120 (69.4%)^b^
	Small intestine^b^	119 (17.9%)^b^	37 (21.5%)^b^
	Colon and rectum	26 (3.9%)	14 (8.1%)
	Others	4 (0.6%)	0 (0%)
Tumor size (median; cm)		4.0 (0.1–35)	5.0 (1.1–25)
Mitosis (median;/50 HPF)		2.6 (0.0–250.0)	5.0 (0.0–250.0)
Symptoms at diagnosis	Yes	257 (38.6%)	^a^
	No	407 (61.2%)	^a^
	Unavailable	1 (0.2%)	^a^
Neoadjuvant	Yes	9 (1.3%)	15 (8.7%)
	No	648 (97.4%)	157 (91.3%)
	Unavailable	8 (1.3%)	0 (0%)
Adjuvant	Yes	37 (5.6%)	30 (17.4%)
	No	627 (94.3%)	142 (82.6%)
	Unavailable	1 (0.1%)	0 (0%)
Surgery	Open	459 (69.0%)	116 (67.4%)
	Laparoscopy	200 (30.1%)	51 (29.7%)
	Local	6 (0.9%)	5 (2.9%)
Completeness of surgery	R0	661 (99.4%)	162 (94.2%)
	R1	4 (0.6%)	10 (5.8%)
Tumor rupture	No	644 (96.8%)	167 (97.1%)
	Yes	21 (3.2%)	5 (2.9%)
	Preoperative	12	3
	Intraoperative	9	2
Histological type	Spindle	538 (80.9%)	83 (48.3%)
	Epithelioid	22 (3.3%)	2 (1.2%)
	Mixed	35 (5.3%)	7 (4.1%)
	Unavailable	70 (10.5%)	80 (46.4%)
Recurrence	No recurrence	570 (87.5%)	124 (72.1%)
	Recurrence	95 (12.5%)	47 (27.9%)
Estimated 5-year RFS (median + SE)		78.6 + 1.8%	72.5 + 3.9%
Overall survival	Alive	600 (90.2%)	151 (87.8%)
	Death	65 (9.8%)	21 (12.2%)
Estimated 5-year OS (median + SE)		91.5 + 1.2%	92.2 + 2.3%

Median follow-up: 4.67 years for study cohort and 5.12 years for validation cohort

*RFS* recurrence-free survival, *OS* overall survival, *SE* standard error

^a^Not available in validation cohort

^b^One duplicated patient with gastric and small intestinal GISTs in each cohort

A questionnaire to participating surgeons indicated the following results (Supplementary Table 1): most surgeons considered that tumor fracture and perforation, piecemeal resection, open biopsy, and macroscopic injuries to the pseudocapsule exposing tumor cells represented rupture; in contrast, they considered that core and needle biopsy without complications, luminal perforation of tumors, peritoneal tumor penetration, and microscopic injuries to the pseudocapsule did not represent rupture.

Tumor size was larger and mitotic count was higher in ruptured GISTs than in nonruptured tumors in the study cohort (Table 2). Patients with ruptured GIST were more symptomatic at admission, regardless of preoperative or intraoperative rupture (Table 2, Supplementary Table 3). Among the nine patients who had neoadjuvant therapy, one patient experienced tumor rupture during surgery. Incidence of rupture was not different between open and laparoscopic surgery in either group. Location (gastric and nongastric) was not correlated with GIST rupture in either the study or validation cohort. There was no significant difference in terms of location, symptoms, tumor size, mitotic count, or recurrence between preoperative and intraoperative rupture (Supplementary Table 2).

**Table 2:** Background of GIST patients with and without tumor rupture (study cohort)

		Nonruptured (*N* = 644)	Ruptured (*N* = 21)	*P* value
Age (years)		66 (18–93)	68 (55–90)	0.2236
Gender	Male	326 (50.6%)	13 (61.9%)	0.3087
	Female	318 (49.4%)	8 (38.1%)	
Primary location	Gastric	493 (76.6%)	13 (61.9%)	0.1215
	Nongastric	151 (23.4%)	8 (38.1%)	
Association of cancer	No	518 (80.4%)	18 (85.7%)	0.7098
	Yes	110 (17.1%)	3 (14.3%)	
	Unavailable	16 (2.5%)	0 (0%)	
Median tumor size (cm)		4.0 (0.1–35.0)	9.6 (2.6–30.0)	0.0008
Symptoms	No	406 (63.0%)	1 (4.7%)	< 0.0001
	Yes	237 (36.8%)	20 (95.2%)	
	Unavailable	1 (0.2%)	0 (0%)	
Neoadjuvant	No	629 (97.7%)	19 (90.5%)	0.1561
	Yes	8 (1.2%)	1 (5%)	
	Unavailable	7 (1.1%)	1 (5%)	
Adjuvant therapy	No	613 (95.2%)	14 (66.7%)	< 0.0001
	Yes	30 (4.7%)	7 (23.3%)	
	Unavailable	1 (0.1%)	0 (0%)	
Surgery	Open	441 (68.5%)	18 (85.7%)	0.2383
	Laparoscopic	197 (30.6%)	3 (14.3%)	
	Local	6 (0.9%)	0 (0%)	
R	R0	642 (99.7%)	19 (90.5%)	< 0.0001
	R1	2 (0.3%)	2 (9.5%)	
Median mitosis (/50 HPF)		2.5 (0.0–250)	13.0 (0.0–115)	0.0004
Cell type	Spindle	518 (80.4%)	20 (95.2%)	0.3347
	Epithelioid	22 (3.4%)	0 (0%)	
	Mixed	35 (5.4%)	0 (0%)	
	Unavailable	69 (10.7%)	1 (4.8%)	
Median RFS	(95% CI; years)	8.4 (8.0–8.9)	2.4 (1.4–3.4)	< 0.0001
Estimated 5-year RFS (median + SE)		80.7 + 1.7%	16.4 + 8.6%	
Recurrence	No	565 (87.7%)	5 (23.8%)	< 0.0001
	Yes	79 (12.3%)	16 (76.2%)	
Recurrence site^a^	Liver	53 (67.1%)^b^	6 (37.5%)^b^	0.0108
	Lung	2 (2.5%)^b^	0 (0%)^b^	
	Local	8 (10.1%)^b^	4 (25%)^b^	
	Peritoneum	24 (30.4%)^b^	14 (87.5%)^b^	
Median OS	(95% CI; years)	11.9 (10.7–13.0)	6.4 (5.6–7.3)	0.0218
Estimated 5-year OS (median + SE)		88.9 + 7.4%	91.6 + 1.2%	
Overall survival	Alive	584 (90.7%)	16 (76.2%)	0.0452
	Dead	60 (9.3%)	5 (23.8%)	
	Death due to GIST	21 (35%)^c^	5 (100%)^c^	
	Death due to other diseases	39 (65%)^c^	0 (0%)^c^	

*RFS* recurrence-free survival, *OS* overall survival, *SE* standard error, *CI* confidence interval

^a^Duplicated number

^b^% of total recurrence in each group

^c^% of total death in each group

**Fig. 1:**
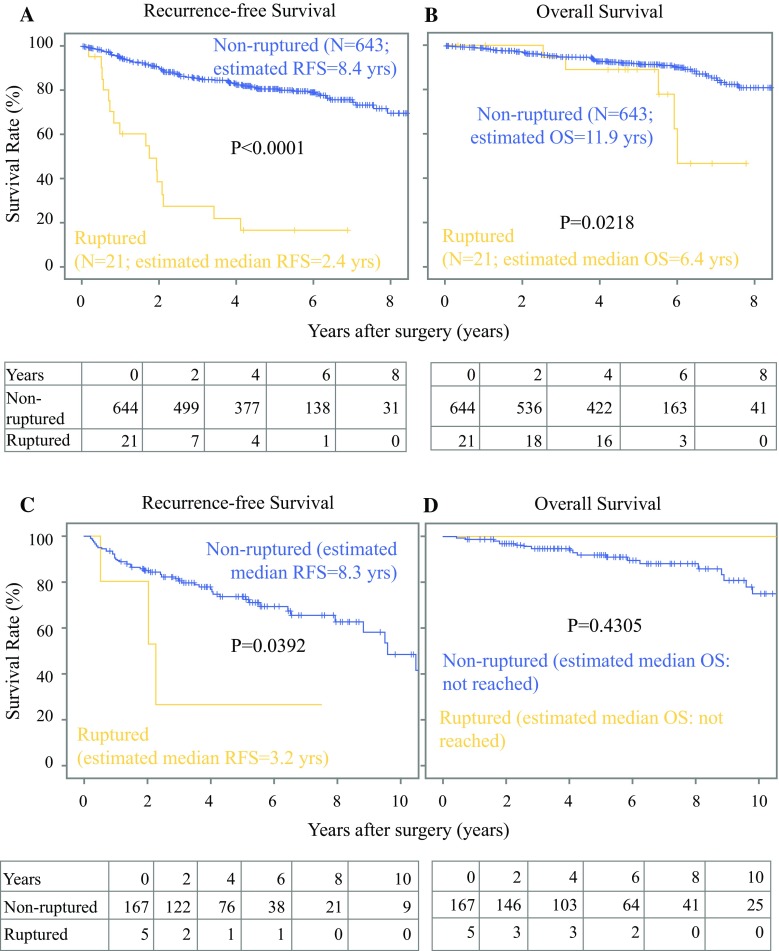
Recurrence-free (**a**, **c**) and overall survival (**b**, **d**) after surgery for patients with or without tumor rupture in the study (**a**, **b**) and validation cohort (**c**, **d**)

**Fig. 2:**
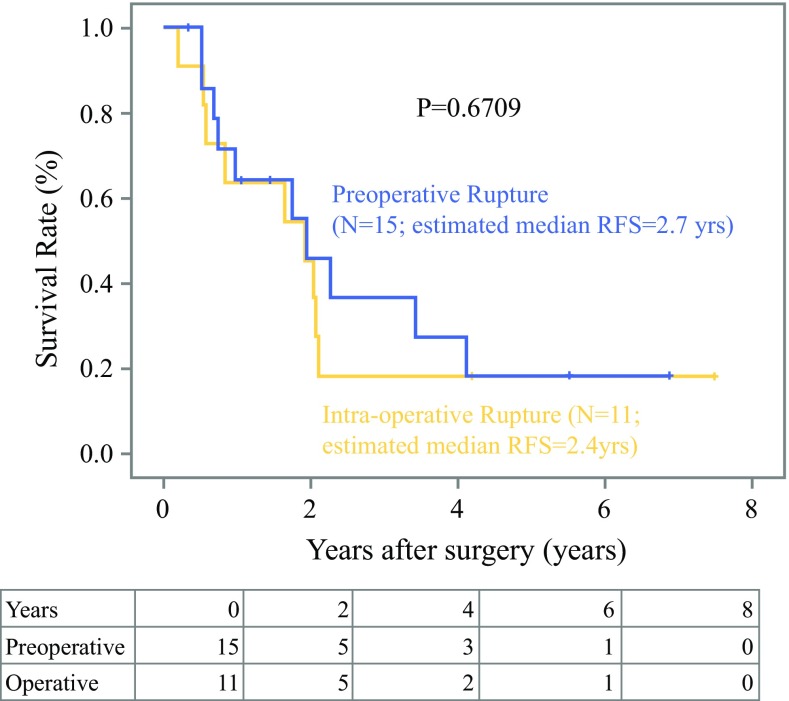
Recurrence-free survival in patients with pre- or intraoperative rupture in the study cohort; there is no significant difference between them

During median follow-up of 4.67 years, there were 95 (12.5%) relapses and 65 (9.8%) deaths in the study cohort, compared with 47 (27.9%) recurrences and 21 (12.2%) deaths in the validation cohort with median follow-up of 5.12 years. Recurrence was more frequent for patients with ruptured GISTs than those without rupture in both cohorts (Table 2, Supplementary Table 4). Median RFS of patients with ruptured GIST [2.4 years; 95% confidence interval (CI) 1.4–3.4 years in the study cohort; *P* < 0.0001, and 3.2 years in the validation cohort; 95% CI 1.3–5.8 years; *P* = 0.0392] was significantly shorter than that of patients with nonruptured GIST (8.4 years; 95% CI 8.0–8.8 years, and 8.4 years; 95% CI 7.4–9.3 years) in the study and validation cohort, respectively (Fig. 1). Cumulative incidence analysis of the study cohort indicated that all events were recurrence of GIST in the rupture group, whereas one-third of events were recurrence of GIST, and deaths due to other diseases might account for the other two-thirds in the nonrupture group (Supplementary Fig. 2). RFS of patients with intraoperative ruptured GIST was not different from that of patients with preoperative rupture (Fig. 2; *P* = 0.6709). In the study cohort, recurrence in patients with ruptured GIST was more frequent in the peritoneum and local lesions compared with those in patients with nonruptured GIST (Table 2). Median OS of patients with ruptured GIST (6.4 years; 95% CI 5.5–7.3 years) was significantly shorter than that of those with nonruptured GIST in the study cohort (11.9 years; 95% CI 10.7–13.0 years; *P* = 0.0218); this was not confirmed in the validation cohort, likely because of the low statistical power and higher rate of imatinib adjuvant therapy.

Multivariate analyses using the Cox proportional hazards model indicated that location, tumor size, mitotic count, and rupture were independent prognostic factors for RFS and that age, gender, and mitotic count were independent prognostic factors for OS in the study cohort (Table 3). In the validation cohort, independent prognostic factors for recurrence included tumor size, mitotic count, rupture, and use of adjuvant therapy, and those for OS were gender, tumor size, and mitotic count (Supplementary Table 4). In the combined analysis of both cohorts, tumor size, mitotic count, location, rupture, and gender were independent prognostic factors for RFS, and age, gender, and mitotic count were independent prognostic factors for OS.

**Table 3:** Multivariate analysis for RFS and OS (study cohort)

Independent prognostic factor	HR (95% CI)	*P* value
Recurrence-free survival (study cohort):		
Location (Ref: gastric)	1.637 (1.339–2.002)	0.0140
Size (cm)	1.070 (1.055–1.085)	< 0.0001
Mitotic count (/50 HPF)	1.012 (1.010–1.014)	< 0.0001
Rupture (Ref: nonrupture)	4.545 (3.307–6.234)	< 0.0001
Overall survival (study cohort):		
Age (years)	1.033 (1.018–1.047)	0.0168
Gender (Ref: female)	2.347 (1.738–3.168)	0.0045
Mitotic count (/50 HPF)	1.014 (1.011–1.017)	< 0.0001

Other factors included in the analysis for RFS using a forward stepwise Cox proportional hazards model were age (*P* = 0.157), gender (*P *= 0.086), symptoms (*P* = 0.551), association of NF1 (*P* = 0.733), neoadjuvant therapy (*P* = 0.454), adjuvant therapy (*P* = 0.453), histology (*P* = 0.177), and R (completeness of surgery) (*P* = 0.887)

Other factors included in the analysis for OS were rupture (*P* = 0.251), tumor location (*P* = 0.743), symptoms (*P* = 0.475), association of NF1 (*P* = 0.311), neoadjuvant therapy (*P* = 0.821), adjuvant therapy (*P* = 0.637), histology (*P* = 0.696), and R (completeness of surgery) (*P* = 0.763)

## Discussion

This study found that ruptured GIST was seen in nearly 3% of primary GISTs, being more symptomatic and exhibiting aggressive features of larger size and higher mitotic count compared with nonruptured tumors. The reported frequency of tumor rupture varies depending on the study; population-based studies indicated that it was less than 10% (1%,^7^ 4.0%,^10^ 5.9%,^3^ and 7.1% 11), while in clinical trials, it was higher than 10% (20% of high-risk GISTs,^17^ 11% of intermediate- and high-risk GISTs,^18^ and 17%^24^). The true incidence of tumor rupture is speculated to be several percent in clinical practice. Ruptured GISTs were shown to be more symptomatic with high-risk features, including larger tumor size and higher mitotic count, and were treated by emergency surgery in previous, as well as present, studies.^11^,^14^,^15^

Tumor rupture occurred both before and during surgery. The frequency of preoperative and intraoperative rupture was similar in this registry study.^17^,^24^ Clinicopathological features and prognostic outcomes of GISTs with preoperative rupture were similar to those with intraoperative rupture, although the sample size was small (Table 2, Supplementary Table 3). The laparoscopic approach was not considered to increase incidence of rupture, nor did neoadjuvant therapy in this study (Table 2, Supplementary Table 3). Taken together, rupture might occur in GISTs with high-risk features regardless of rupture timing; intraoperative rupture might not be due to surgical techniques but rather due to tumor factors, such as fragility, size, and/or adhesion to adjacent organs.

Tumor rupture may result in peritoneal seeding of tumor cells, hence surgery may be considered R1 even if achieving macroscopic complete resection. This study, however, revealed that some surgeons considered such surgery to be R0 after macroscopic complete resection during the study period (Table 2, Supplementary Table 3). Tumor rupture was an independent prognostic factor for both RFS and OS before imatinib.^25^ We showed that rupture remains an important prognostic factor of RFS, but not OS, in the era of imatinib. This is likely due to the activity of imatinib, sunitinib, and/or regorafenib used following recurrence. In fact, most patients received imatinib and subsequently sunitinib after recurrence, although patients receiving adjuvant therapy represented a small fraction. All guidelines suggest that patients with high-risk GISTs should have adjuvant therapy, but only 7 of 21 patients (33%) with ruptured GIST received adjuvant therapy in this study. This low rate of adjuvant therapy reflects the historical background of the registries. Taken together, tumor rupture is an independent prognostic factor of recurrence, but not OS, after complete resection in the era of imatinib.

The definition of tumor rupture was subjectively determined and was not yet agreed upon when patients registered in this study underwent surgery. However, the survey showed that the results appeared to be similar to the definition of tumor rupture recently proposed by Holmebakk et al.^14^ There are some differences; half of surgeons did not consider microscopic infiltration into neighboring structures as tumor rupture when they performed en bloc resection, while macroscopic injury to the pseudocapsule was viewed as tumor rupture in this study. In our definition of tumor rupture, there was no prognostic difference between preoperative and intraoperative rupture, suggesting that our definition might be acceptable.

There are some limitations to consider. The study is retrospective, and the number of patients was limited, especially in the events of recurrence and death; however, it is based on two multi-institutional registry studies. The use of two different cohorts may help to check reproducibility to confirm the obtained results. The median follow-up of the registry studies was 4.7 and 5.1 years for the study and validation cohort, respectively, which may be insufficient to evaluate OS in the era of imatinib, although it may be long enough to determine RFS. Historically, imatinib has been the standard therapy for recurrent diseases. However, adjuvant therapy was not used sufficiently; thus, adjuvant imatinib was not an independent prognostic factor as previously suggested.^26^ Finally, the definition of tumor rupture was subjective among surgeons; however, as mentioned above, participating investigators shared similar views regarding tumor rupture, indicating that this multi-institutional study is valid.

This study focused on ruptured GISTs. Tumor rupture may be defined by tumor fracture and perforation at the tumor site, piecemeal resection, open biopsy, and macroscopic injuries to the pseudocapsule, whereas core and needle biopsy without complications, luminal perforation of tumors, microscopic peritoneal breaks on tumors, or microscopic breaks of the pseudocapsule on pathological examination are not considered to be tumor rupture. By this definition, GISTs with tumor rupture were seen in several percent of GISTs in clinical practice, showed aggressive features of larger size and higher mitotic count regardless of time of rupture, and had poor prognosis even in the era of imatinib; nevertheless, general standard oncologic principle of avoiding surgical tumor rupture is critically important in surgery. To improve the prognosis of patients with ruptured GISTs, more prolonged imatinib adjuvant therapy may be required.

## Electronic supplementary material

Below is the link to the electronic supplementary material.

Supplementary material 1 (PDF 345 kb)
