# Supplementary material for: Clinicopathological Features and Prognosis of Primary GISTs with Tumor Rupture in the Real World
Source: Ann Surg Oncol. 2018 May 11;25(7):1961–9. doi: 10.1245/s10434-018-6505-7 (PMC5976711; doi:10.1245/s10434-018-6505-7)

Supplement Table 1. The results of the questionnaire for primary surgeon of each participant hospitals

**Conditions**	**Yes**	**No**	**Yes/No**	**Pre or Intra**[#]
Tumor fracture with blood-tinged ascites at laparotomy	25	0	0	Pre
Intestinal perforation on tumor with blood-tinged ascites at laparotomy	23	2	0	Pre
Microscopically direct tumor invasion into adjacent resected organs	10	15	0	Pre
Intraluminal tumor perforation	3	22	0	Pre
Piecemeal resection during operation	25	0	0	Intra
Iatrogenic fracture of tumors	25	0	0	Intra
Macroscopic injuries to the pseudocapsule exposing tumor cells into the peritoneal cavity	20	4	1	Intra
Minor or microscopic injuries to pseudocapsule without exposure of tumor cells	3	22	0	Intra
Open biopsy	21	4	0	Intra
Core needle biopsy without complications	7	18	0	Pre
Fine needle biopsy without complications	1	24	0	Pre

The questionnaire was sent to the 33 hospitals and institutions, and the answers were received from 25 primary investigators in each hospital and institution. (Valid Response Rate：76％)
In the questionnaire, it was asked for investigators whether each listed item may be considered as tumor rupture. Yes: investigators consider that it is rupture; No: investigators do not consider; Yes/No: depending on circumstances and they had no clear answer.
# indicates classification of preoperative or intraoperative rupture in the study; Pre: preoperative rupture, Intra: intraoperative rupture

Supplement Table 2. Comparison of between the two tumor rupture types (study cohort)

		Preoperative (N=12)	Intraoperative (N=9)	P value
Primary Location	Gastric	9 (75%)	4 (44%)	0.1536
	Non-gastric	3 (25%)	5 (56%)	
Median tumor size (cm)		7.5 (2.6-30.0)	10.6 (4.0-30.0)	0.7872
Symptoms	No	0 (0%)	1 (11%)	0.2367
	Yes	12 (100%)	8 (89%)	
Median Mitosis (/50 HPF)		2.5 (0.0-250)	16.6 (1.0-75.0)	0.2898
Recurrence	No	4 (33%)	1 (11%)	0.2367
	Yes	8 (67%)	8 (89%)	
Survival	Alive	10 (83%)	6 (67%)	0.3749
	Dead	2 (17%)	3 (33%)	

Supplement Table 3. Backgrounds of GIST patients with and without tumor rupture (Validation cohort)

		Non-ruptured (N=167)	Ruptured (N=5)	P value
Age (years)		62 (33-89)	75 (17-81)	<0.0001
Gender	Male	99 (59%)	1 (20%)	0.1624
	Female	68 (41%)	4 (80%)	
Primary Location#	Gastric	116 (69%)	3 (60%)	0.6442
	Non-gastric	51 (31%)	2 (40%)	
Association of cancer	No	130 (78%)	5 (100%)	0.5861
	Yes	37 (22%)	0 (0%)	
	Unavailable	0 (0%)	0 (0%)	
Median tumor size (cm)		5.0 (1.1-25.0)	5.0 (3.3-19.0)	0.4027
Neoadjuvant#	No	152 (91%)	5 (100%)	1.0000
	Yes	15 (9%)	0 (0%)	
	Unavailable	0 (0%)	0 (0%)	
Adjuvant therapy#	No	138 (83%)	4 (80%)	1.0000
	Yes	29 (17%)	1 (20%)	
	Unavailable	0 (0%)	0 (0%)	
Surgery	Open	112 (67%)	4 (80%)	0.0336
	Laparoscopic	51 (31%)	0 (0%)	
	Local	4 (2%)	1 (20%)	
R	R0	161 (96%)	1 (20%)	<0.0001
	R1	6 (4%)	4 (80%)	
Median mitosis (/50 HPF)		5 (0-250)	7 (4-50)	0.6353
Cell type	Spindle	79 (47%)	4 (80%)	0.7991
	Epithelioid	2 (1%)	0 (0%)	
	Mixed	7 (4%)	0 (0%)	
	Unavailable	79 (47%)	1 (20%)	
Median RFS	(95% CI; years)	8.4 (7.4-9.3)	3.2 (0.7-5.8)	0.0392
Estimated median 5-year RFS (median+SE)		73.7%±3.9%	26.7%±22.6%	
Recurrence	No	122 (73%)	2 (40%)	0.1333
	Yes	45 (27%)	3 (60%)	
Recurrence sites#	Liver	26 (58%)	2 (66%)	0.0392
	Lung	1 (2%)	2 (66%)	
	Local	5 (11%)	1 (33%)	
	Peritoneum	15 (33%)	1 (33%)	
	Others	4 (9%)	0 (0%)	
	Unavailable	1 (2%)	0 (0%)	
Estimated 5-year median OS (median+SE)$		91.9%±2.4%	100%	
Survival	Alive	146 (87%)	5 (100%)	1.0000
	Dead	21 (13%)	0 (0%)	
	Death due to GIST	17 (81%)	0 (0%)	
	Death due to other diseases	4 (19%)	0 (0%)	

#: duplicated number

$: Median OS could not be calculated.

Supplement Table 4. Multivariate analysis for RFS and OS (Validation cohort and integrated analysis)

Recurrence-free Survival

Independent Prognostic Factors	HR (95%CI)	P value
Size (cm)	1.013 (1.006 - 1.020)	0.0001
Mitotic Count (/50HPF)	1.020 (1.012 - 1.027)	<0.0001
Rupture (Ref: non-rupture)	12.08 (2.957 – 49.32)	0.0015

Other factors included in the analysis using a forward stepwise Cox proportional hazards model are age (P=0.318), gender (P=0.299), tumor location (P=0.469), neoadjuvant therapy (P=0.813), adjuvant therapy (P=0.0879; HR=0.409), and R (completeness of surgery) (P=0.293).

Overall Survival

Independent Prognostic Factors	HR (95%CI)	P value
Gender (Ref: female)	7.258 (1.222 – 43.10)	0.02919
Mitotic Count (/50HPF)	1.013 (1.003 - 1.024)	0.01330
Size (cm)	1.014 (1.002 – 1.025)	0.01999

Other factors included in the analysis using a forward stepwise Cox proportional hazards model are age (P=0.779), rupture (P=0.591), tumor location (P=0.717), neoadjuvant therapy (P=0.529), adjuvant therapy (P=0.3523) and R (completeness of surgery) (P=0.368).

The results of integrated analysis of the two data sets.

Recurrence-free Survival

Independent Prognostic Factors	HR	95%CI		P value
Gender	1.393	1.178	1.645	0.047
Rupture	6.539	4.998	8.525	<0.001
Tumor Size	1.006	1.004	1.008	0.003
Mitotic Count	1.013	1.011	1.015	<0.001
Location	1.495	1.254	1.782	0.022

Other factors included in the analysis using a forward stepwise Cox proportional hazards model are age (P=0.498), R (P=0.851), neoadjuvant therapy (P=0.943), and adjuvant therapy (P=0.126).

Overall Survival

Independent Prognostic Factors	HR	95%CI		P value
Age	1.025	1.013	1.038	0.031
Gender	2.445	1.859	3.216	0.001
Mitotic Count	1.012	1.001	1.015	<0.001

Other factors included in the analysis using a forward stepwise Cox proportional hazards model are

rupture (P=0.402), tumor size (P=0.206), R (P=0.245), tumor location (P=0.592), neoadjuvant therapy (P=0.646), and adjuvant therapy (P=0.4406).

Supplement Figure 1. Patient selection (Study cohort)

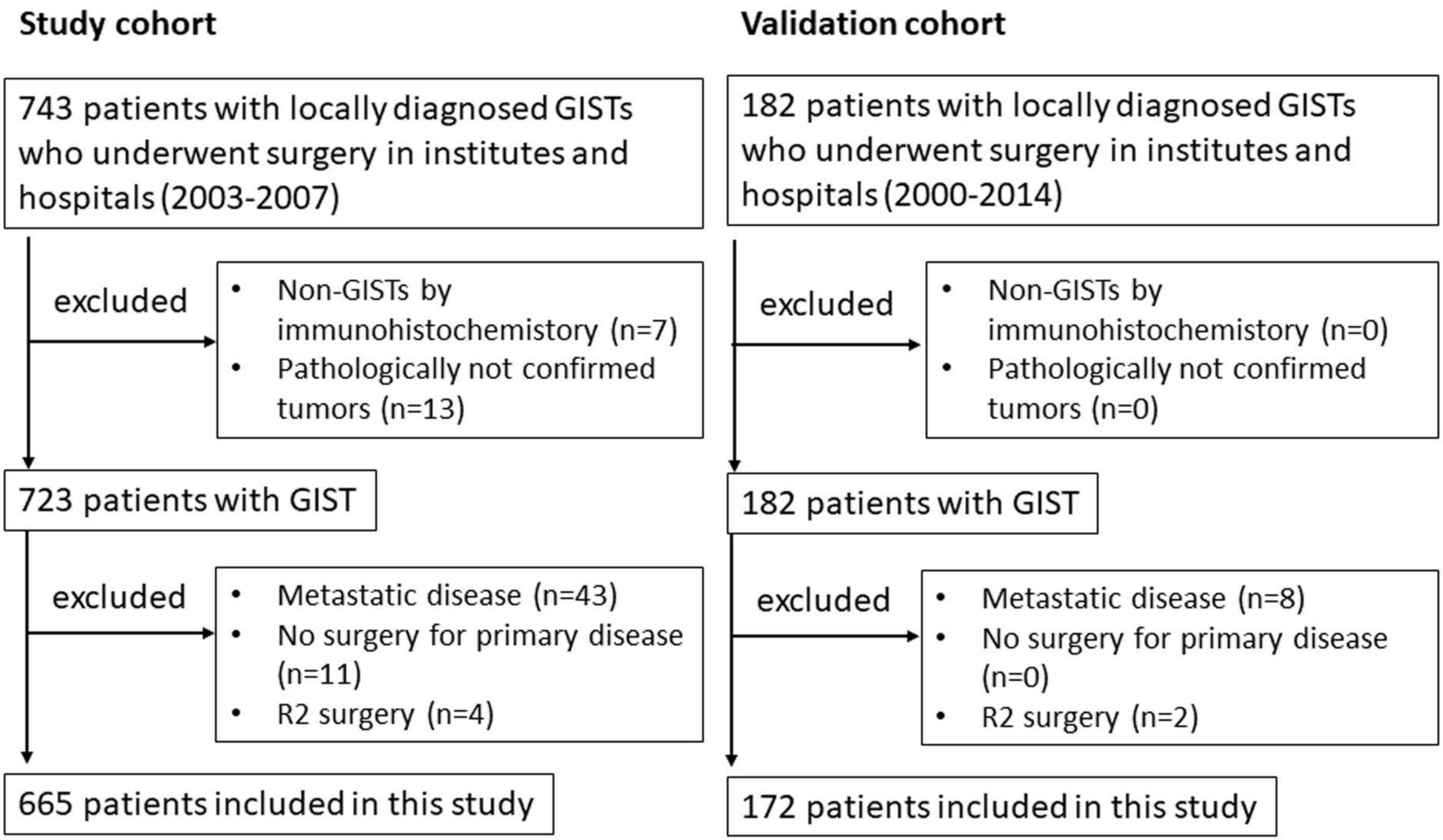

Supplement Figure 2. Cumulative Incidence of Recurrence (Study Cohort)

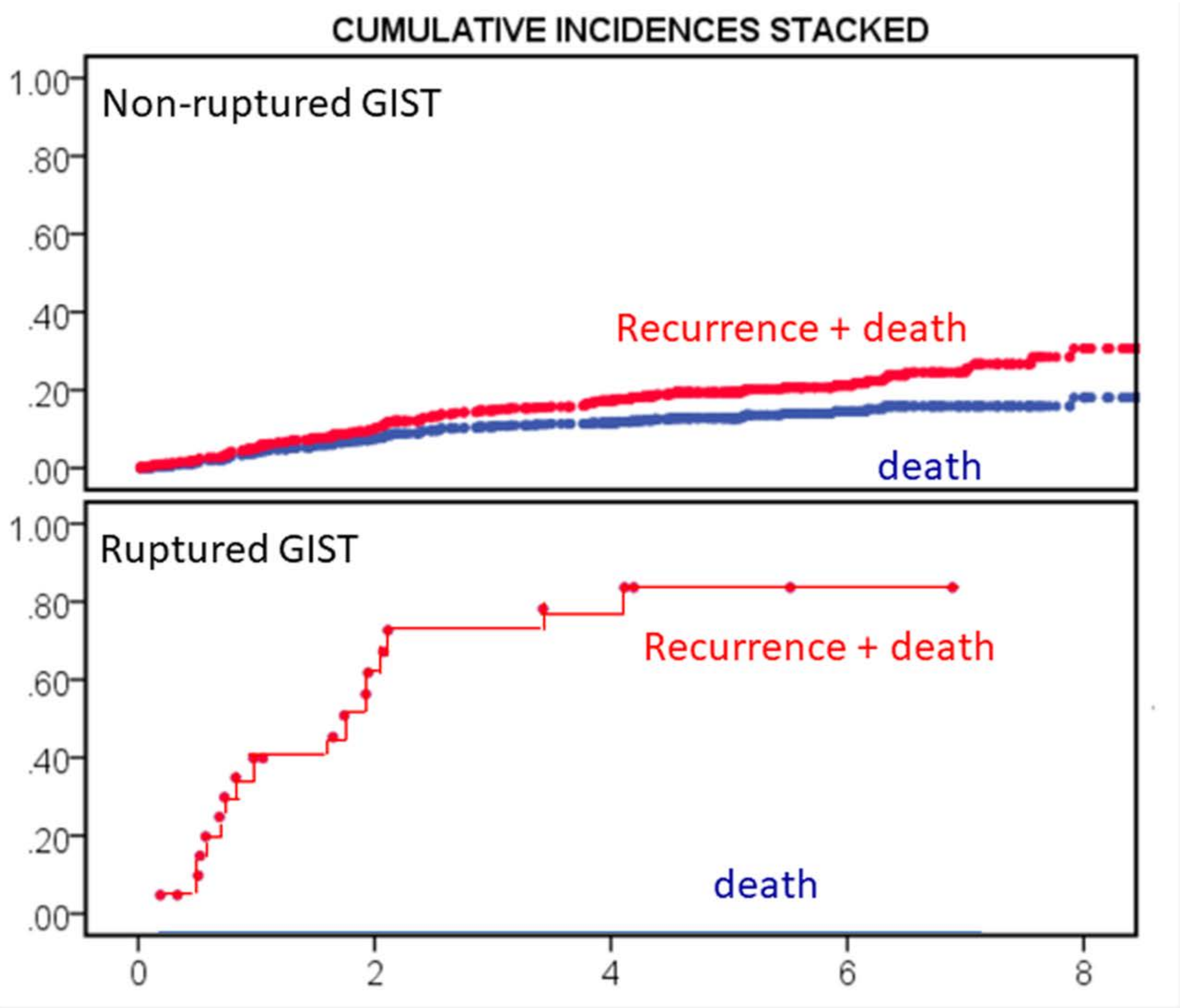

Supplement: Supplementary file 1 — Supplementary material 1 (PDF 345 kb) [file 10434_2018_6505_MOESM1_ESM.pdf]
